# Molecular characteristics of erythromycin-resistant *Streptococcus pneumoniae* from pediatric patients younger than five years in Beijing, 2010

**DOI:** 10.1186/1471-2180-12-228

**Published:** 2012-10-09

**Authors:** Lin Zhou, Xiang Ma, Wei Gao, Kai-hu Yao, A-dong Shen, Sang-jie Yu, Yong-hong Yang

**Affiliations:** 1 Key Laboratory of Major Diseases in Children and National Key Discipline of Pediatrics (Capital Medical University), Ministry of Education, Beijing Pediatric Research Institute, Beijing Children’s Hospital, Capital Medical University, No. 56 Nan-li-shi Road, Beijing, 100045, China

## Abstract

**Background:**

*Streptococcus pneumoniae* is the main pathogen that causes respiratory infections in children younger than five years. The increasing incidence of macrolide- and tetracycline-resistant pneumococci among children has been a serious problem in China for many years. The molecular characteristics of erythromycin-resistant pneumococcal isolates that were collected from pediatric patients younger than five years in Beijing in 2010 were analyzed in this study.

**Results:**

A total of 140 pneumococcal isolates were collected. The resistance rates of all isolates to erythromycin and tetracycline were 96.4% and 79.3%, respectively. Of the 135 erythromycin-resistant pneumococci, 91.1% were non-susceptible to tetracycline. In addition, 30.4% of the erythromycin-resistant isolates expressed both the *ermB* and *mef* genes, whereas 69.6% expressed the *ermB* gene but not the *mef* gene. Up to 98.5% of the resistant isolates exhibited the cMLS_B_ phenotype, and Tn*6002* was the most common transposon present in approximately 56.3% of the resistant isolates, followed by Tn*2010*, with a proportion of 28.9%. The dominant sequence types (STs) in all erythromycin-resistant *S. pneumoniae* were ST271 (11.9%), ST81 (8.9%), ST876 (8.9%), and ST320 (6.7%), whereas the prevailing serotypes were 19F (19.3%), 23F (9.6%), 14 (9.6%), 15 (8.9%), and 6A (7.4%). The 7-valent pneumococcal conjugate vaccine (PCV7) and 13-valent pneumococcal conjugate vaccine (PCV13) coverage of the erythromycin-resistant pneumococci among the children younger than five years were 45.2% and 62.2%, respectively. ST320 and serotype 19A pneumococci were common in children aged 0 to 2 years. CC271 was the most frequent clonal complex (CC), which accounts for 24.4% of all erythromycin-resistant isolates.

**Conclusions:**

The non-invasive *S. pneumoniae* in children younger than five years in Beijing presented high and significant resistance rates to erythromycin and tetracycline. The expressions of *ermB* and *tetM* genes were the main factors that influence pneumococcal resistance to erythromycin and tetracycline, respectively. Majority of the erythromycin-resistant non-invasive isolates exhibited the cMLS_B_ phenotype and carried the *ermB*, *tetM*, *xis*, and *int* genes, suggesting the spread of the transposons of the Tn*916* family. PCV13 provided higher serotype coverage in the childhood pneumococcal diseases caused by the erythromycin-resistant isolates better than PCV7. Further long-term surveys are required to monitor the molecular characteristics of the erythromycin-resistant *S. pneumoniae* in children.

## Background

*Streptococcus pneumoniae*, the main pathogen that causes respiratory infections in children younger than five years, can cause various pneumococcal diseases, such as otitis media, sinusitis, pneumonia, meningitis, pleurisy, and bacteremia. Pneumococcal disease has been a major public health problem worldwide. In 2005, the World Health Organization (WHO) estimated that 1.6 million people die of pneumococcal diseases annually, of which the deaths of 0.7 million to 1 million were children younger than five years [1].

Antibiotics are often the first treatment of choice for pneumococcal infections. However, the increasing resistance of *S. pneumoniae* to various antibiotics, including macrolides and tetracyclines, makes pneumococcal infections difficult to treat especially in children and in regions like China. The resistance rate of *S. pneumoniae* to erythromycin and to tetracycline among children younger than five years in Beijing ranged from 87% to 94% and above 80%, respectively [2]. Pneumococcal macrolide resistance is mediated by two major mechanisms, namely, target modification by a ribosomal methylase encoded by the *ermB* gene and drug efflux encoded by the *mef* gene. In *S. pneumoniae*, the tetracycline resistance is a result of the acquisition of one of the two genes, *tetM* or *tetO*, both of which encode ribosome protection proteins [3,4]. Pneumococcal resistance to erythromycin and tetracycline is frequently associated with the insertion of the *ermB* gene into the transposons of the Tn*916* or Tn*917* family (Tn*6002*, Tn*2010*, Tn*3872*, Tn*1545*, and Tn*6003*) that contains the *tetM* gene.

Resistant-clonal isolates are distributed in different countries and regions, which results in the spread of bacterial resistance. The molecular epidemiological monitoring network (http://spneumoniae.mlst.net/pmen/) has published 43 international clones of *S. pneumoniae*, among which the clones of serotypes 6A, 6B, 14, 15A, 19A, 19F, 23F, and 35B were found to be associated with bacterial resistance. Thus, a study on the molecular epidemiology of *S. pneumoniae* for children in one region is beneficial to monitor pneumococal-resistant clones.

Studies on the characteristics of erythromycin-resistant *S. pneumoniae* in China are rare. Thus, the present study focuses on analyzing the phenotypic and genotypic characteristics of erythromycin-resistant pneumococcal isolates from pediatric patients in Beijing in 2010 as well as their respective relationships.

## Methods

### Bacterial isolates

A total of 140 *S. pneumoniae* isolates were collected from the nasopharyngeal swabs of pediatric patients younger than five years with upper respiratory infections in the Beijing Children’s Hospital in 2010 after their parents or legal guardians have given their consent. The study was approved by the Ethics Committee of the Beijing Children’s Hospital, and all procedures were performed in accordance with the Helsinki Declaration [5]. The isolates were identified based on the typical colony morphology, Gram staining, optochin sensitivity test (Oxoid Company, Britain), and Omni serum assay (Statens Serum Institut, Copenhagen, Denmark). Only one isolate was selected from the same subject. Of the 140 *S. pneumoniae* isolates, 57 were obtained from pediatric patients aged 0 to 2 years (≤2 years old) and 83 from those aged 2 years to 5 years (>2 but ≤5 years old).

### Antibiotic susceptibility testing

The E-test (AB Biodisk, Sweden) method was performed to determine the antibiotic susceptibility of the 140 pneumococcal isolates to erythromycin and tetracycline according to the guidelines established by the Clinical and Laboratory Standards Institute (CLSI). The CLSI 2010 criteria [6] for minimum inhibitory concentrations (MICs) were applied to classify the susceptible, intermediate, or resistant isolates with the following breakpoints: erythromycin, ≤0.25 μg/mL, 0.5 μg/mL, and ≥1 μg/mL; and tetracycline, ≤2 μg/mL, 4 μg/mL, and ≥8 μg/mL, respectively. ATCC49619 was used as the quality control strain and was included in each set of tests to ensure accurate results.

### Macrolide resistance phenotype

Macrolide resistance phenotyping was performed via double-disk diffusion using standard disks of erythromycin (15 μg) and clindamycin (2 μg) (Oxoid Company, Britain). A blunting of the clindamycin inhibition zone adjacent to the erythromycin disk (“D zone”) indicated the presence of the inducible macrolide-resistant phenotype (iMLS_B_), whereas the absence of blunting indicated the presence of the constitutive macrolide-resistant phenotype (cMLS_B_). The M macrolide phenotype was characterized by clindamycin susceptibility with no blunting of the inhibition zone around the clindamycin disk.

### DNA extraction

Chromosomal DNA was isolated from the overnight cultures of the isolates that were grown on 5% trypticase soy agar by using the DNA Mini Kit (SBS Genetech, Beijing) according to the manufacturer’s instructions.

### Detection of genes and related transposons

The macrolide-resistance genes *ermB* and *mef* were detected using polymerase chain reaction (PCR) with oligonucleotide primers specific for each gene as described in the previous studies [7]. The PCR products of the *mef* genes were digested with *BamHI* to distinguish the *mefA* and *mefE* gene subclasses [8].

The Tn*916* and Tn*917* transposon-related genes (*int*, *xis*, *tnpA*, and *tnpR*), the tetracycline-resistance gene *tetM*, and the promoter of the *aph3’-III* gene were detected by PCR using the primers described in previous studies [9-13]. The resistance gene combinations related to the different presumed transposons were Tn*6002* (*ermB*, *tetM*, *int*, and *xis*), Tn*2010* (*ermB*, *tetM*, *int*, *xis*, and *mefE*), Tn*3872* (*ermB*, *tetM*, *tnpA*, and *tnpR*), Tn*1545*, or Tn*6003* (*ermB*, *tetM*, *int*, *xis*, and *aph3’-III*).

### Multi locus sequence typing (MLST)

The housekeeping genes *aroE*, *gdh*, *gki*, *recP*, *spi*, *xpt*, and *ddl* were amplified via PCR [14]. The sequences of each of the seven loci were compared with those of all known alleles at the loci as well as with the STs in the database of the pneumococcal multi locus sequence typing (MLST) website (http://spneumoniae.mlst.net). New allelic numbers or new ST numbers were assigned by the curator of the pneumococcal MLST website. The eBURST v3 software (http://spneumoniae.mlst.net/eburst/) was used to investigate the relationships between the isolates and to assign a clonal complex (CC) based on the stringent group definition of six out of seven shared alleles.

### Serotyping

Pneumococcal serotyping was performed through the Quellung reaction by using Pneumotest kits and type-specific antisera (Statens Serum Institut, Copenhagen, Denmark) for the erythromycin-resistant isolates as previously described [15]. The isolates that reacted negatively were non-typeable. The PCV7 and PCV13 coverage was estimated by calculating the percentage of isolates that expressed the serotypes included in the vaccine.

### Statistical analysis

The data from the antibiotic susceptibility testing were set up and analyzed using the WHONET 5.3 software, which was recommended by the WHO. The *χ*^2^-test and the Fisher’s accurate probability tests were performed using the SPSS version 13.0 software to compare proportions. Differences with *P* < 0.05 were considered statistically significant.

## Results

### Antibiotic susceptibility

The susceptibility and MICs to erythromycin and tetracycline of 140 pneumococcal isolates that were collected among children of different ages are presented in Table 1. Based on the CLSI 2010 criteria, the resistance rate of all isolates to erythromycin was 96.4% (135/140), whereas the susceptibility rate was merely 2.9% (4/140). Up to 98.5% (133/135) of the erythromycin-resistant pneumococcal isolates exhibited high MICs (>256 μg/mL). The erythromycin resistance rates between children aged 0 to 2 years and 2 to 5 years were all above 94.0%, with 54 and 81 isolates, respectively. No significant difference was found between the two age groups (*P* > 0.05). The total resistance rate of all the isolates to tetracycline reached 79.3% (111/140). No difference was also found in tetracycline resistance between children aged 0 to 2 years and 2 to 5 years (*P* > 0.05). A total of 110 (78.6%) isolates were resistant to both erythromycin and tetracycline, and 91.1% (123/135) of the erythromycin-resistant strains were non-susceptible (intermediate and resistant) to tetracycline.

**Table 1:** **Susceptibility and minimum inhibitory concentrations (MICs) of 140 *S. pneumoniae *isolates to erythromycin and tetracycline**

**Age group**	**No.**	**Antibiotics**	**Susceptible**	**Intermediate**	**Resistant**	**MIC**_**50**_**(μg/mL)**	**MIC**_**90**_**(μg/mL)**	**MIC range (μg/mL)**
0 to 2 years	57	erythromycin	3 (5.3%)	0 (0%)	54 (94.7%)	>256	>256	0.125- > 256
		tetracycline	9 (15.8%)	5 (8.8%)	43 (75.4%)	12	16	0.064-16
2 to 5 years	83	erythromycin	1 (1.2%)	1 (1.2%)	81 (97.6%)	>256	>256	0.125- > 256
		tetracycline	6 (7.3%)	9 (10.8%)	68 (81.9%)	12	16	0.094-32
0 to 5 years	140	erythromycin	4 (2.9%)	1 (0.7%)	135 (96.4%)	>256	>256	0.125- > 256
		tetracycline	15 (10.7%)	14 (10.0%)	111 (79.3%)	12	16	0.064-32

### Detection of resistance genes and phenotype

All 135 erythromycin-resistant isolates were detected for macrolide- and tetracycline-resistance genes as well as macrolide-resistant phenotype. Almost 30.4% isolates expressed both the *ermB* and *mef* genes, whereas 69.6% were positive for the *ermB* gene but negative for the *mef* gene. The resistant isolates had no different carrying proportions of both the *ermB* and *mef* genes, as well as only *ermB*, between the two aforementioned pediatric age groups (*P* > 0.05) (Table 2). All *mef*-positive isolates carried the *mefE* gene. Among the erythromycin-resistant pneumococcal isolates, all the 123 tetracycline-resistant and intermediate isolates carried the *tetM* gene. However, eight of the 12 tetracycline-susceptible isolates carried the *tetM* gene. Up to 98.5% (133/135) of the resistant isolates exhibited the cMLS_B_ phenotype, but only two isolates expressed the M phenotype. No iMLS_B_ phenotype was found among the resistant isolates.

**Table 2:** Detection of erythromycin-resistance genes for 135 erythromycin-resistant pneumococcal isolates

**Macrolide-resistance genes**		**No. (%)**	**Age group**		**MICs (μg/mL) distribution (No.)**			**MIC range (μg/mL)**
***ermB***	***mef***		**0 to 2 years**	**2 to 5 years**	**3**	**12**	**>256**	
+	+	41 (30.4%)	18 (13.3%)	23 (17.1%)	1	1	39	3- > 256
+	-	94 (69.6%)	36 (26.7%)	58 (42.9%)			94	>256

### Transposon distribution

**Figure 1:**
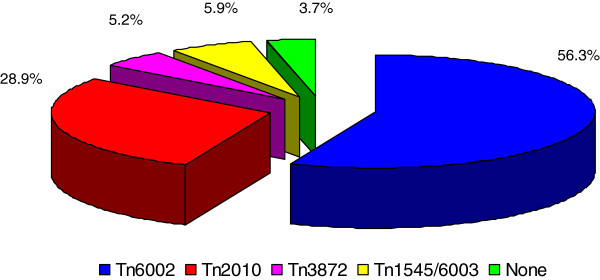
**Distribution of Tn*916*- and Tn*917*-related transposons in the 135 erythromycin-resistant pneumococcal isolates.**

Among the 135 erythromycin-resistant pneumococci, 76 isolates (56.3%) contained *ermB*, *tetM*, *int*, and *xis* genes related to Tn*6002*. 39 isolates (28.9%) were detected for the presence of *ermB*, *tetM*, *int*, *xis*, and *mefE* genes, carrying the transposon of Tn*2010*. Seven isolates (5.2%) were positive for the *ermB*, *tetM*, *tnpA*, and *tnpR* genes related to Tn*3872*. Eight isolates (5.9%) containing the *ermB*, *tetM*, *int*, and *xis* genes were also positive for the promoter of the *aph3’-III* gene related to Tn*1545*/*6003* via PCR, of which only two isolates had the *mefE* gene. The *int*, *xis*, *tnpA*, *tnpR*, *aph3’-III*, and *mefE* genes were not detected in the remaining five isolates (3.7%) (Figure 1).

### Multi locus sequence typing

**Figure 2:**
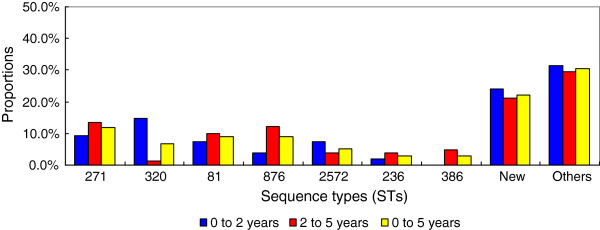
Distribution of sequence types (STs) with age in the 135 erythromycin-resistant pneumococcal isolates.

**Figure 3:**
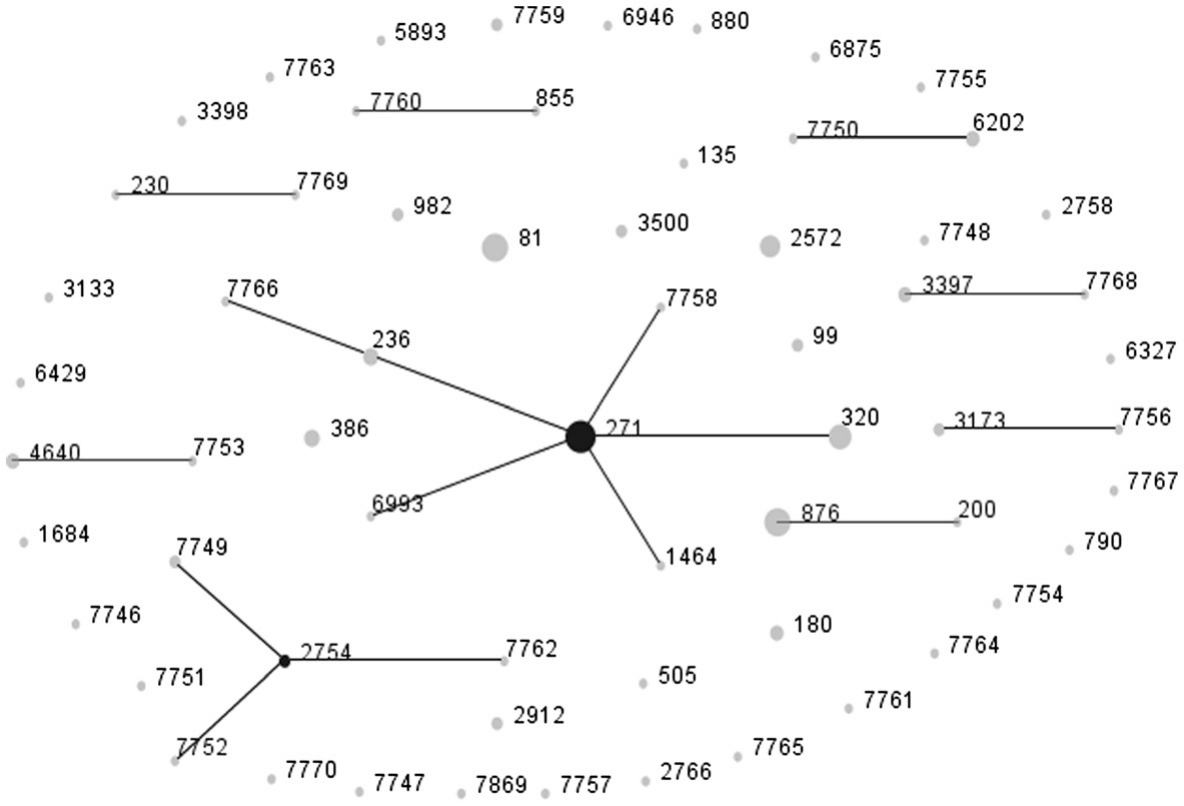
**Population snapshot of 135 erythromycin-resistant pneumococcal isolates as revealed by eBURST analysis.** One spot indicates one ST. The size of one spot corresponds to the number of pneumococcal isolates with the same ST. The lines indicate the presence of single locus variant SLV links among particular STs.

A total of 62 STs were found in the erythromycin-resistant *S. pneumoniae*, of which 28 STs were newly assigned, via MLST analysis. Of the new STs, 19 types were novel combinations of known alleles (ST6875, ST6946, and ST7746 to ST7762). Up to 9 profiles (ST7763 to ST7770 and ST7869) contained 10 new alleles, namely, *aroE*236, *gdh*353, *gki*353, *gki*354, *gki*355, *recP*207, *recP*208, *spi*332, *spi*338, and *ddl*512. The four predominant STs of all resistant pneumococci were ST271 (11.9%, 16/135), ST81 (8.9%, 12/135), ST876 (8.9%, 12/135), and ST320 (6.7%, 9/135) (Figure 2). Of the common STs, the proportion of ST320 was higher among children aged 0 to 2 years than that of the other age group (*P* < 0.05). However, the percentage of the other STs, such as ST81, ST236, ST271, ST876, ST386, and ST2572, did not show any difference between the two age groups (*P* > 0.05). The eBURST analysis results showed 9 CCs and 36 singletons (Figure 3). CC271 was the most frequent CC, with a proportion of 24.4% (33/135) among the resistant isolates.

### Serotyping and vaccine coverage

**Figure 4:**
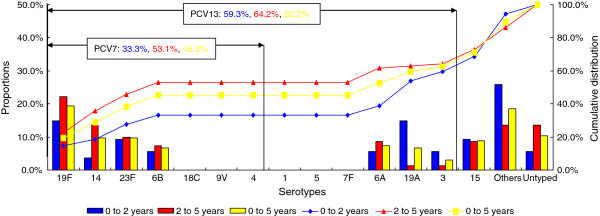
Serotype distribution and vaccine coverage with age among the 135 erythromycin-resistant pneumococcal isolates.

Among the 135 erythromycin-resistant pneumococci, 121 isolates (89.6%) could be serotyped, of which the prevailing five serotypes were 19F (19.3%), 23F (9.6%), 14 (9.6%), 15 (8.9%), and 6A (7.4%), which accounted for 54.8% (74/135). The pneumococcal isolates of serotype 19A were significantly common among children aged 0 to 2 years than that of 2 to 5 years (*P* < 0.05). However, the pneumococcal isolates of the other serotypes were not different between the two age groups (*P* > 0.05). The PCV13 coverage for the erythromycin-resistant isolates was 62.2% (84/135). This value was higher than that of PCV7 (45.2%, 61/135) among all children younger than five years as well as the children aged 0 to 2 years (*P* < 0.05). The PCV7 coverage of children aged 2 to 5 years was significantly higher than that of 0 to 2 years (*P* < 0.05). However, no difference in PCV13 coverage was observed among these two age groups (*P* > 0.05) (Figure 4).

### Relations of sequence types, serotypes, resistance genes, and transposons

Several associations were observed between the STs, serotypes, macrolide-resistance genes, as well as Tn*916*- and Tn*917*-related transposons for the erythromycin-resistant pneumococcal isolates (Table 3). The dominant ST of the serotype 19F isolates was ST271, followed by ST236. On the other hand, that of the serotype 14, 23F, and 6B isolates was ST876, ST81, and ST386, respectively. The ST of all the serotype 19A pneumococci was ST320. All isolates of CC271, which was identified as serotype 19F and 19A, carried two macrolide-resistance genes, *ermB* and *mefE*. However, the *mefE* gene was not found among the isolates of other CCs, such as CC2754, CC230, CC3173, CC3397, CC6202, and CC855. Tn*6002* was distributed among the isolates of seven CCs except for CC271 and CC3173, among which the dominant transposons were Tn*2010* and Tn*3872*, respectively. Tn*1545*/*6003* was found in the isolates of ST180, ST271, ST320, ST505, ST2572, ST7759, ST7760, and ST7768.

**Table 3:** Sequence types, serotypes, macrolide-resistance genes, and transposons for 135 erythromycin-resistant pneumococci

**Clonal complex**	**ST**	**NO.**	**Serotype (no.)**	**Resistance genes (no.)**		**Transposons (no.)**				
				***ermB***	***ermB*** **+** ***mefE***	**Tn*6002***	**Tn*2010***	**Tn*3872***	**Tn*1545*/*6003***	**None**
CC271	271	16	19F (16)		16		15		1	
	320	9	19A (9)		9		8		1	
	236	4	19F (4)		4		4			
	1464	1	19F (1)		1		1			
	6993	1	19F (1)		1		1			
	7758	1	19F (1)		1		1			
	7766	1	19F (1)		1		1			
CC2754	2754	2	6A (1), untyped (1)	2		2				
	7749	2	13/28 (2)	2		2				
	7752	1	untyped (1)	1		1				
	7762	1	6B (1)	1		1				
CC200	200	1	14 (1)	1		1				
	876	12	14 (11), 19F (1)	10	2	9	2	1		
CC230	230	1	non-23F (1)	1		1				
	7769	1	non-7A/F (1)	1		1				
CC3173	3173	2	6A (2)	2				2		
	7756	1	6B (1)	1				1		
CC3397	3397	3	15 (3)	3		3				
	7768	1	15 (1)	1					1	
CC6202	6202	3	15 (3)	3						3
	7750	1	15 (1)	1		1				
CC855	855	1	6A (1)	1		1				
	7760	1	6A (1)	1					1	
CC4640	4640	3	untyped (3)	3		3				
	7753	1	untyped (1)		1		1			
Singletons	81	12	23F (11), non-23F (1)	11	1	11	1			
	386	4	6B (4)	4		4				
	2572	7	non-23F (6), 24/31/40 (1)	6	1	5	1		1	
	180	3	3 (3)	3		2			1	
	2912	2	6A (2)	2		2				
	3500	2	8 (2)	2		2				
	7759	2	untyped (2)	2		1			1	
	982	2	6A (2)	2		2				
	99	2	11A/C/D (1), 16/36/37 (1)	2		2				
	OTs^a^	28	untyped (6), others^b^ (22)	25	3	19	3	3	1	2
Total		135		94	41	76	39	7	8	5

^a^ Other sequence types. Only one isolate was identified as each of the other STs.

^b^ Other serotypes included 3 (1), 6A (1), 6B (3), non-7A/F (1), 10B/C/F (1), non-12B/F (1), 14 (1), 15 (4), non-17F (1), non-19A/C/F (1), 19F (1), 23F (2), 13/28 (2), 21/39 (1), 24/31/40 (1).

## Discussion

*S. pneumoniae* is a Gram-positive encapsulated diplococcus and is often transmitted by direct contact with respiratory secretions from the infected and healthy carriers. The disease develops by contiguous spread to the sinuses or the middle ear, aspiration into the lower respiratory tract, which causes pneumonia, or by invasion into the bloodstream with or without the seeding of secondary sites. According to the 2000 Chinese census data, an estimated 260,768 (113,000 to 582,382) pneumonia and 902 (114 to 4,463) meningitis cases were caused by *S. pneumoniae*. The estimated deaths from *S. pneumoniae* pneumonia and meningitis were 10,703 (4,638 to 23,904) and 75 (10 to 370), respectively [16]. This result indicates that pneumococcal diseases are a significant health problem among children in China.

Pneumococcal resistance to antimicrobials, such as macrolides and tetracyclines, is a serious and rapidly growing problem worldwide. In the Asian Network for Surveillance of Resistant Pathogen study, Song et al. [17] demonstrated that Asian regions had the highest levels of *S. pneumoniae* antibiotic resistance. A total of 59.3% of erythromycin-resistant pneumococcal isolates were observed in Asian countries. Vietnam had the highest resistance rate, followed by Taiwan, Korea, Hong Kong, and the hinterlands of China [18]. The erythromycin resistance rate of *S. pneumoniae* in 1998 to 1999 in Beijing was 71.0% [19], which was much higher than that in 1996 to 1997 [18]. Pneumococci data of hospitalized pediatric patients with respiratory infections showed 85.7% erythromycin resistance in Shanghai [20] and 92.1% in Chongqing [21]. In the present study, the erythromycin resistance rate of *S. pneumoniae* was higher at 96.4%, and most of the isolates had high MICs (>256 μg/mL), which indicated an increasing trend of pneumococcal erythromycin resistance in the hinterlands of China. Geographical variations were observed in the phenotypic and genotypic characteristics of erythromycin-resistant *S. pneumoniae*. The *ermB* gene was the most common mechanism for erythromycin resistance in the hinterlands of China, Taiwan, Sri Lanka, and Korea, similar to the results of this study for the children in Beijing. However, the *mef* gene was more common in Hong Kong, Singapore, Thailand, and Malaysia [18]. In Europe, the *ermB* gene was the dominant macrolide-resistance gene, especially in France, Spain, Switzerland, and Poland. On the other hand, the *mef* gene was common in Greece and Germany [22]. In the present study, the MLS_B_ phenotype was the predominant phenotype among the erythromycin-resistant pneumococcal isolates, which was in accordance with previous studies in China [23,24]. However, the M phenotype was more prevalent than the MLS_B_ phenotype in other countries, such as in Canada and in the United Kingdom [9,25].

The resistance of *S. pneumoniae* to tetracycline was also significantly high in China, which was similar to that of erythromycin. This result may be related to the abuse of tetracycline in agriculture and edible animals. A multi-center research on the antibiotic resistance of *S. pneumoniae* involving four cities in China revealed that 82.1% of pneumococcal isolates were tetracycline-resistant among 1-month-old to 5-year-old children with acute upper respiratory infections [23]. The tetracycline non-susceptible rate among the invasive erythromycin-resistant pneumococcal isolates collected in Australia was 75.5% [26]. This value was lower than the non-invasive erythromycin-resistant isolates in the current study. The present study, in addition to previous ones [10,11,27], proved that the *tetM* gene was responsible for tetracycline resistance in *S. pneumoniae*. In the present study, we found that the eight pneumococcal isolates with the *tetM* gene were susceptible to tetracycline. Amezaga et al. [9] identified a 10 bp deletion in the sequence of the *tetM* gene of one tetracycline-susceptible isolate. This result was relative to the *tetM* sequence in tetracycline-resistant isolates. Thus, further studies are necessary.

Tetracycline resistance is associated with erythromycin resistance in pneumococcal isolates, which are transmitted by the transposons of the Tn*916* or Tn*917* family including Tn*6002*, Tn*2010*, Tn*3872*, Tn*1545*, and Tn*6003*. Tn*6002*, which was first detected in *Streptococcus cristatus*, originated from the insertion of an *ermB*-containing DNA fragment into Tn*916*, which carries the *tetM* gene [28,29]. Our study showed that Tn*6002* was more common among erythromycin-resistant isolates in Beijing children, which accounts for 56.3%. In addition, Tn*2010* is a composite element of adding the *mefE* gene on the basis of Tn*6002*, with a proportion of 28.9% in the present study. Tn*3872* results from the insertion of the *ermB*-containing Tn*917* transposon [30] into Tn*916*[31]. Tn*1545* and Tn*6003* have similar compositions; they both contain the kanamycin resistance gene *aph3’-III* aside from the erythromycin- and tetracycline-resistance determinants *ermB* and *tetM*. In this study, the transposons Tn*3872* and Tn*1545*/Tn*6003* were rare at approximately 11.1%, indicating that Tn*3872* and Tn*1545*/Tn*6003* were not the main factors for erythromycin and tetracycline resistance in Beijing children. Moreover, we also found five pneumococcal isolates without transposon determinants that carried the *ermB* and *tetM* genes or only *ermB* gene. Further studies are necessary to verify if these five isolates contain unknown transposons.

Three conjugate vaccines, namely, PCV7, PCV10, and PCV13, were introduced to prevent pneumococcal infections in children. PCV13 included serotypes 1, 3, 5, 6A, 7F, and 19A plus the PCV7 serotypes 4, 6B, 9V, 14, 18C, 19F, and 23F. In this study, the serotypes 23F, 19F, 14, and 6B were common among *S. pneumoniae* from Beijing children younger than five years. This result was similar with the previous studies in China [20,32,33], but different from that of the other European countries, in which the serotypes 1, 3, 6A, 7F, and 19A were common among pneumococcal isolates [34]. Since the introduction of PCV7, the incidence of pneumococcal disease because of PCV7-serotypes has significantly declined in many countries. However, several countries have reported an increased rate of pneumococcal disease in non-PCV7 serotypes. This phenomenon, termed ‘replacement’, is associated with specific pneumococcal serotypes or clones [35]. In China, the PCV7-serotypes were more popular among children for two reasons: first, PCV7 has been on the market for only four years in China since 2008. Second, only about 1% of Chinese children use PCV7 for their routine pneumococcal immunization. We found that the PCV13 coverage of the erythromycin-resistant isolates was higher than that of PCV7 among all children younger than five years as well as the children aged 0 to 2 years because of the high rates of serotypes 3, 6A, and 19A. Moreover, the PCV7 coverage of children aged 2 to 5 years was also significant higher than that of children aged 0 to 2 years. All these results indicate that PCV13 controls the pneumococcal diseases caused by the erythromycin-resistant isolates better than PCV7 for children, especially those younger than two years.

Maiden et al. [36] introduced the MLST approach to monitor the epidemiology of bacteria based on multi locus enzyme electrophoresis. Enright and Spratt were the first to apply MLST for pneumococcal studies [14]. The MLST database enables the global sharing of data and helps in the exchange of data between different laboratories. Thus, it can be used to monitor the molecular epidemiology of *S. pneumoniae* worldwide. In the present study, the prevalent STs were ST271, ST81, ST876, and ST320. In Shanghai, ST236 and ST271 were the most common STs for *S. pneumoniae*[37]. ST320, ST271, and ST876 were the prevalent types among the invasive pneumococcal isolates collected from 11 cities in China [38]. In Norway, the frequent STs were ST199, ST176, and ST36 among the isolates collected from the children attending daycare centers [39]. Several associations were found between STs, serotypes, and macrolide-resistance genes in this study. The dominant STs of the serotype 19F, 14, 23F, and 6B isolates were ST271, ST876, ST81, and ST386, respectively. ST320 was more common in children aged 0 to 2 years than in other age groups and all were from the serotype 19A pneumococci. Notably, ST320 was found to be the predominant type among pneumococcal serotype 19A isolates from ten Asian countries [40]. This suggests that ST320 has an important function in pneumococcal diseases in children. The ST320 clone of serotype 19A is expected to be more prevalent worldwide because of the wide use of PCV7. A systematic study showed that Taiwan19F-14 was one of the two dominant clones for erythromycin-resistant isolates in Asian regions [41]. Taiwan19F-14 (ST236), a multidrug-resistant pneumococcal molecular epidemiology network clone and one of the most main clones causing invasive pneumococcal diseases in Asian countries [42], was associated with seven STs in this study, ST236, ST271, ST320, ST1464, ST6993, ST7758, and ST7766. ST236 is a single locus variant of ST271 and a double locus variant of ST320. According to eBURST analysis, both ST271 and ST320 belong to CC271, which was the most common CC observed in this study. CC271 emerged in the United States after the introduction of PCV7, and expressed both the *ermB* and *mef* genes [41], as shown in the present study.

## Conclusions

*S. pneumoniae* in children younger than five years in Beijing presented high and significant resistance rates to erythromycin and tetracycline. The *ermB* and *tetM* genes were the main factors for pneumococcal erythromycin and tetracycline resistance, respectively. Majority of the erythromycin-resistant isolates exhibited the cMLS_B_ phenotype and carries the *ermB*, *tetM*, *xis*, and *int* genes, which suggested the spread of the transposons of the Tn*916* family. PCV13 provided higher serotype coverage in the childhood pneumococcal diseases caused by the erythromycin-resistant isolates better than PCV7. The incidence of erythromycin-resistant *S. pneumoniae* among children is continuously increasing; thus, further long-term studies of their molecular characteristics are necessary.

## Authors’ contributions

LZ and XM conducted the laboratory work, performed the analysis, wrote the draft, and are the co-first authors for the same contributions of this study. WG, KY, AS, and SY provided the bacterial isolates and laboratory supplies. YY planned the study. All authors read and approved the final manuscript.
